# Cobalt-Doped Bioactive Glasses for Biomedical Applications: A Review

**DOI:** 10.3390/ma16144994

**Published:** 2023-07-14

**Authors:** Francesco Baino, Maziar Montazerian, Enrica Verné

**Affiliations:** 1 Institute of Materials Physics and Engineering, Department of Applied Science and Technology, Politecnico di Torino, 10129 Torino, Italy; enrica.verne@polito.it; 2 Northeastern Laboratory for Evaluation and Development of Biomaterial (CERTBIO), Federal University of Campina Grande, Campina Grande 58429-900, PB, Brazil; maziar_montaz@yahoo.com; 3 Department of Materials Science and Engineering, The Pennsylvania State University, University Park, State College, PA 16801, USA

**Keywords:** biomaterials, bioglass, therapeutic ion, osteogenesis, angiogenesis

## Abstract

Improving angiogenesis is the key to the success of most regenerative medicine approaches. However, how and to which extent this may be performed is still a challenge. In this regard, cobalt (Co)-doped bioactive glasses show promise being able to combine the traditional bioactivity of these materials (especially bone-bonding and osteo-stimulatory properties) with the pro-angiogenic effect associated with the release of cobalt. Although the use and local delivery of Co^2+^ ions into the body have raised some concerns about the possible toxic effects on living cells and tissues, important biological improvements have been highlighted both in vitro and in vivo. This review aims at providing a comprehensive overview of Co-releasing glasses, which find biomedical applications as various products, including micro- and nanoparticles, composites in combination with biocompatible polymers, fibers and porous scaffolds. Therapeutic applications in the field of bone repair, wound healing and cancer treatment are discussed in the light of existing experimental evidence along with the open issues ahead.

## 1. Introduction

Over the past half-century, bioactive glasses have been increasingly investigated as implantable biomaterials for a wide range of medical applications, ranging from bone repair to cancer treatment [1,2]. Since their discovery in 1969 [3], bioactive glasses have been typically proposed for bone restoration given their physico-mechanical affinity with the hard tissues in the body; in recent years, some special glass compositions have shown great promise in contact with soft tissues and organs [4,5].

Bioactive glasses exhibit a well-known bone-bonding capability by forming a surface layer of hydroxyapatite upon contact with biofluids in vivo or after incubation in simulated body fluids in vitro—and this is actually referred to as the “traditional” bioactivity of these biomaterials—while simultaneously supporting some key regenerative processes, such as angiogenesis and osteogenesis during their dissolution [6,7].

Hench et al. used the ternary Na_2_O–CaO–SiO_2_ phase diagram to develop the first bioactive glass, named “45S5 Bioglass^®^” (composition: 45SiO_2_–24.5CaO–24.5Na_2_O–6P_2_O_5_ wt.%) in the effort to better treat limb amputations and osseous defects of soldiers coming back from the Vietnam War to the USA [8,9]. This glass was FDA-approved for clinical use in 1985, and since then, it has been applied to >2 million patients worldwide, mainly for dental and orthopedic restorations [10,11].

Over the years, many other biomedical glass compositions have been designed, investigated and proposed for clinical use [12], and moreover, some of them were reported to induce antibacterial effects [13,14] and/or induce anti-inflammatory responses [15,16]. In general, the addition of glass network modifiers has significant effects on the material’s overall properties, including biological effects.

In this regard, two important points deserve to be highlighted: (i) The traditional concept of bioactivity, associated with bone-bonding ability, has been progressively overcome and has expanded its meaning to include other therapeutic functions, not necessarily limited to interactions with bone; (ii) these therapeutic effects are primarily dictated by the ionic dissolution products released from bioactive glasses—hence, designing glass composition is the key to tailor the overall “glass bioactivity” [17].

Multiple metallic ions have been incorporated in bioactive glasses to develop multifunctional systems for tackling multiple therapeutic challenges simultaneously (e.g., osteogenesis + angiogenesis, osteogenesis + treatment of infections, etc.). Furthermore, the release of biologically active ions has not only an obvious impact on human health and metabolic/cellular processes but could be a valuable alternative to expensive and potentially toxic pharmaceuticals [18]. This latter advantage is of particular significance in the fight against resistant bacterial strains, which may become insensitive to antibiotics but are effectively killed or inactivated by antimicrobial ions such as silver [19].

Significant research has been carried out about the incorporation of alkaline, alkaline-earth and transition metal ions in bioactive glasses, with a special focus on their biological effect, as summarized in some comprehensive reviews [20,21,22]. Just to mention the roles played by the most commonly investigated dopants, strontium exerts antiresorptive/anti-osteoporotic effects; silver, gallium, zinc and copper exhibit antibacterial properties (the last also elicit a pro-angiogenic effect); and magnesium and fluoride have an osteo-stimulatory effect, the latter preventing the formation of dental caries, too [23]. Pantulap et al. [24] also discussed the role of some exotic elements on the properties of bioactive glasses, which can be useful not only to improve therapeutic performances but also to impart extra-functionalities such as fluorescence, luminescence and radiation shielding that have great importance from a diagnostic viewpoint, thus yielding advanced biomaterials for theranostic applications.

**Figure 1:**
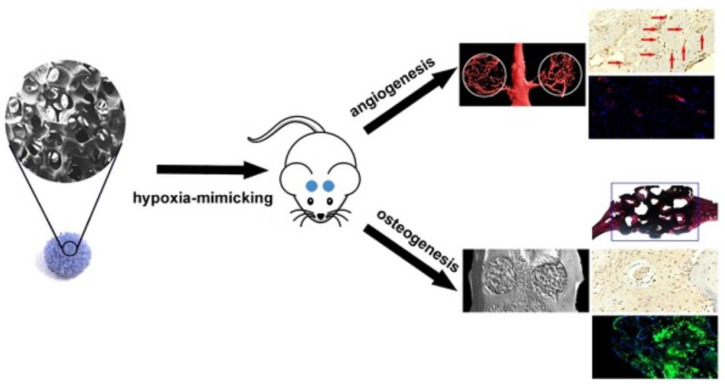
Schematic presentation of cobalt-releasing bioactive glasses as biomaterials that mimic hypoxia, promote angiogenesis and enhance osteogenesis. Reproduced from [26] under a CC-BY license.

Cobalt is an attractive dopant for bioactive glasses that have shown promise for both hard and soft tissue engineering applications. According to a recent report [24], cobalt has been the 9th most commonly investigated dopant for bioactive glasses over the past 20 years (about 50 scientific publications) after strontium (around 225 articles), zinc (170 articles), silver (160 articles), magnesium (140 articles), copper (125 articles), iron (90 articles), boron (80 articles) and titanium (65 articles). In Figure 1, a schematic demonstration showcases the proposal of cobalt-releasing bioactive glasses as biomaterials that mimic hypoxia. Co-doped bioactive glasses are intended for the artificial stabilization of HIF-1α. The application of these glasses in biomedicine stems from their ability to promote the hypoxia response, cell growth and angiogenesis. This is achieved through the synergistic combination of hypoxia-promoting properties of cobalt and the inherent osteogenic characteristics of bioactive glasses. The enhanced expression of HIF-1α and VEGF further supports this notion, as also discussed in the following Section 2. However, to the best of the authors’ knowledge, no specific review paper dedicated to Co-doped bioactive glasses has been published yet. In order to bridge this gap, we reviewed all the relevant literature published from the first study by Azevedo et al. [25] in 2010 to date and provided a picture of the topic, along with highlighting the limitations and challenges ahead.

## 2. Rationale of Incorporating Cobalt in Bioactive Glasses for Tissue Engineering Applications

Cobalt, as a dopant in glasses, is well known to impart a brilliant blue color to the material, even at a very low dosage. In this regard, Savvova et al. [27] proposed the incorporation of small amounts of CoO (0.03–0.05 wt.%) in bioactive glasses and glass-ceramics as a simple means to improve implant visualization in vivo. However, apart from this very specific “chromatic” application at the frontier between aesthetics and function, the main reason behind the doping of bioactive glasses with cobalt is that Co^2+^ ions, once released into the biofluids, exhibit a potent pro-angiogenic action. Blood supply and capillary/blood vessel growth are key requirements for the healing of vascularized tissues, including bone. In large osseous defects, if angiogenesis is not somehow induced, bone tissue typically grows up to a thickness of just 150–200 µm before facing necrosis, owing to the lack of proper nourishment, which is insufficient to heal most bone defects [28]. Therefore, there have been several efforts to incorporate cobalt—as well as other pro-angiogenic dopants—into the most common and successful biomaterials for bone tissue engineering, i.e., bioactive ceramics and glasses [29], because of the relatively low cost of ion-driven strategies as compared with the administration of exogenous growth factors.

While cobalt can indeed play a potent pro-angiogenic role in vitro and in vivo, its effects on the structure and physico-chemical properties of bioactive glasses are relatively limited. Although cobalt typically acts as a network modifier in glasses, dopant doses below 4 mol.% are too low to cause highly significant changes in the structure of the material [30]; on the other hand, a low dosage is necessary to avoid toxicity. Nevertheless, some macroscopic effects have been reported. Glass density and all the major mechanical properties—including compressive/flexural strength, Young’s/shear modulus and hardness—were reported to increase with increasing cobalt amounts (up to 4 mol.%) in melt-derived 45S5- [31,32] and 13-93-derived glasses [33]. While physico-mechanical effects are clear, the role played by cobalt on glass reactivity is a bit controversial. On the one hand, cobalt was reported to increase the dissolution rate of the glass, suggesting the network to be somewhat chemically weaker [34]; consistently, the apatite-forming ability in vitro was improved by increasing dopant amounts [32]. On the other hand, the presence of cobalt in bioactive glasses was shown to decrease ion release, and the formation of hydroxyapatite in vitro was delayed with CoO addition as well [25].

The biological impact of cobalt is multifaceted and very interesting. Under physiological conditions, angiogenesis does not take place. The hypoxia-inducible factor-1α (HIF-1α) is hydroxylated by prolyl hydroxylase domain (PHD) proteins when ascorbate is present in the cell to counteract the effect of reactive oxide species (ROS). While Fe(II) assists the action of PHD over HIF-a, such as the oxygen present in the cytosol, ROS oxidates it to Fe(III), making it impair the hydroxylation function of the protein complex. Once hydroxylated, the HIF-1α is rapidly degraded by the ubiquitin–proteasome pathway in a manner that depends on an E3 ubiquitin ligase complex containing the von Hippel-Lindau tumor-suppressor protein (VHL); hence, it cannot upregulate VEGF, a signal protein responsible for new blood vessel formation. The scenario changes when hypoxia takes place or environmental conditions vary. Specifically, cobalt has been observed to hinder the ascorbate entry into the cytosol, halting the PHD function, leading to upregulation of HIF-1α and thus increasing the secretion of VEGF. Nevertheless, rising ascorbate levels have been shown to reverse this effect [35]. HIF-1α is responsible for stimulating SOX9, a protein that is positively involved in the production of chondrogenic markers. HIF-1 modulation by Co-doped bioactive glasses was therefore supposed to be of significance in the context of cartilage regeneration [36].

Cobalt has been proven to be ineffective in concentrations below 5 ppm. Above around 10 ppm, instead, recipient cells showed viability reduced by 40% [37]. This result is not surprising because cobalt is known to be carcinogenic, cytotoxic and genotoxic to human cells. Several mechanisms have been proposed to explain its detrimental behavior. One of them is integrin interference for microvascular endothelial cells. Integrins are intermembrane proteins used by the cell to make the cytoskeleton adhere to a substrate. They are involved in a number of biological functions, including cell movement, growth, differentiation, survival and apoptosis signaling. Cobalt was found to induce focal adhesion contact derangement, which easily leads to apoptosis; specifically, cobalt and other bivalent transition metals could bond to integrins’ active sites and impair their physiological functions, as shown in an in vitro study with cells soaked in a 0.7 mM Co solution for 3 days [38].

Another mechanism proposed to explain cobalt toxicity relies on an increased ROS production via a Fenton-like reaction, as follows [39]:

$$\begin{array}{c} Co\left(II\right)+O_{2}^{\cdot -}\to Co\left(I\right)+O_{2}\\ 2O_{2}^{\cdot -}+2H^{+}\to H_{2}O_{2}+O_{2}\\ Co\left(I\right)+H_{2}O_{2}\to Co\left(II\right)+OH^{.}+{OH}^{-} \end{array}$$

This should result in DNA damage, including chromosome aberrations, single and double-strand breaks and sister chromatid exchanges. Nevertheless, some studies suggest an indirect action to elicit DNA damage through inhibition of DNA repair [40]. In this regard, a study using human bone mesenchymal stem cells and 5 mol.% Co-doped bioactive glass showed that such concentration significantly reduced cell viability, hence setting such a threshold for potential use in bone repair [41].

## 3. Processing of Co-Doped Bioactive Glasses and Related Products

Co-doped bioactive glasses have been basically produced by (i) melt-quenching route, which requires the melting of precursor oxide/carbonate powders at high temperature (typically well above 1000 °C) followed by rapid cooling (quenching) of the melt to obtain an amorphous material, or (ii) variants of the sol-gel process to obtain particles with controllable shape and size, also in the nano-range. In this regard, bioactive glass nanoparticles are, in general, particularly appealing in biomedicine because of their unique properties, such as the capability of acting as controlled drug delivery systems (especially if produced in a mesoporous form) or theranostic agents [42,43,44,45].

Although in a few reports, Co-doped bioactive glasses have been studied and used as standalone products, e.g., in the form of particles or wholly glass scaffolds, much other research is addressed to embedding a Co-containing dispersed phase within a polymeric matrix, thereby obtaining composite biomaterials with higher pliability, flexibility, softness and ease of being intraoperatively cut/shaped by surgeons to match the defect anatomy. Incorporation of a Co-releasing material indeed imparts key extra-functionalities to the otherwise non-bioactive polymer, such as pro-angiogenic effect.

In the following subsections, for the purpose of simplicity, Co-doped glasses and relevant composite products are classified according to the method of glass processing (melt-quenching or sol-gel route).

### 3.1. Melt-Derived Glasses

Melt-derived materials were produced in the form of bulk pieces, fibers or powders, which can be further processed to fabricate 3D scaffolds or microfibers. Some examples of porous constructs are displayed in Table 1.

Smith et al. [30] produced Co-doped bulk glasses (cylinders) after pouring the melt into preheated graphite molds. In another study [46], fibers were pulled from the glass melt, collected and subsequently broken into particles with sizes in the range of 100 to 300 μm, which were eventually used for in vitro biological tests with adult stem cells.

As reported by Hoppe et al. [37], adding CoO as a network modifier in melt-derived silicate glasses yields some effects on thermal behavior—which are of great significance from the viewpoint of processing—including decreasing glass transition temperature (T_g_) and increasing crystallization onset (T_x_). As an overall result, the sinterability window becomes larger with increasing content of cobalt that stabilizes the amorphous glass state. This effect of improving glass stability against devitrification induced by cobalt is consistent with the results associated with other network modifiers, primarily some alkaline and alkaline-earth ions, which are commonly added to bioactive glass compositions [47,48,49].

**Figure 2:**
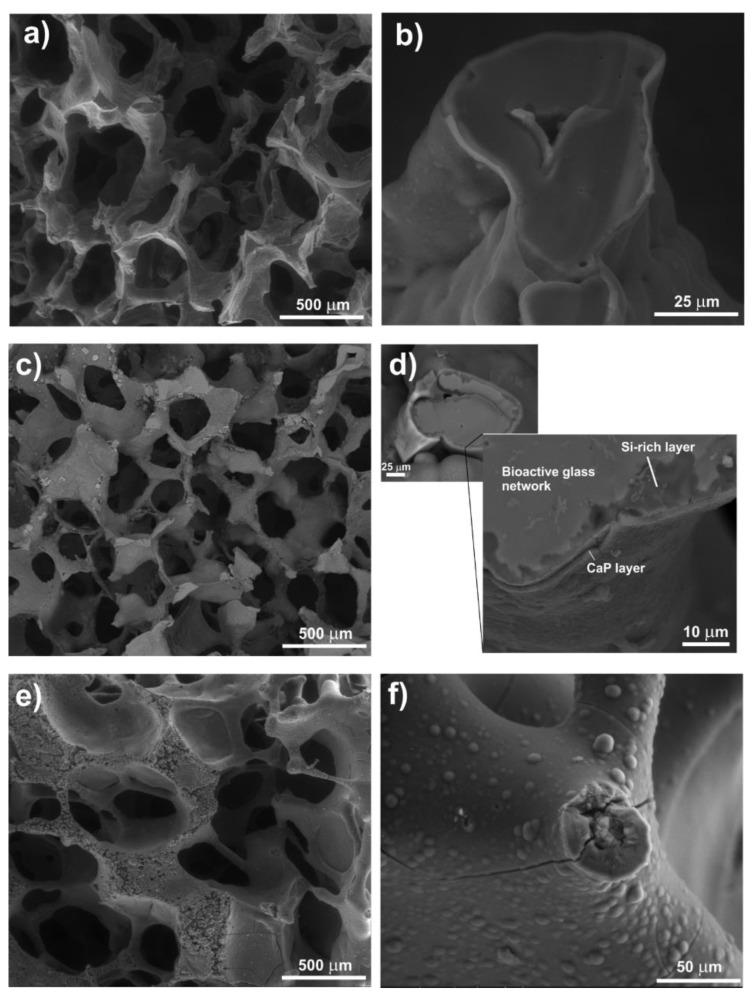
SEM micrographs showing Co-doped 1393-derived foam-like scaffolds after immersion in SBF for 1 day (**a**,**b**), 3 days (**c**,**d**) and 7 days (**e**,**f**) and highlighting the apatite-forming ability of the material. Reproduced from [37] with permission.

Melt-derived powders were used in some studies to fabricate porous scaffolds. The first report dealing with Co-doped bioactive glass scaffolds was published by Prof. Boccaccini’s team [37], who produced Co-doped 13-93 glass foams by the sponge replica method (Figure 2). Incorporation of 1 or 5 wt.% of CoO in the material yielded no significant differences in terms of architecture/morphology and compressive strength compared with 13–93 parent glass scaffolds; however, dissolution rate upon immersion in simulated body fluid (SBF) was accelerated along with the inhibition of crystallization of the surface calcium phosphate layer into hydroxyapatite (it remained in an amorphous state and Ca^2+^ could be locally substituted by Co^2+^ ions).

**Figure 3:**
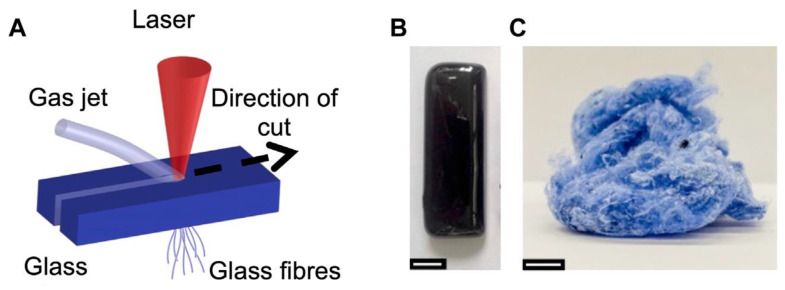
Co-releasing glasses fabricated by laser spinning: (**A**) schematic of the laser spinning process using a glass precursor plate, (**B**) photograph of a bioactive glass precursor plate and (**C**) mesh of fibers produced. Scale bar is 10 mm. Reproduced from [53] under a CC-BY license.

Melt-derived multicomponent Co-containing glass particles were also mixed with polycaprolactone (PCL) to produce extrudable inks for use in the manufacturing of 3D-printed scaffolds [50], with electrospun fish-derived collagen fibers to fabricate fibrous composite mats [51], or with bovine collagen/glycosaminoglycans to obtain ultra-porous composite scaffolds [52]. Successful production of single-phase fibrous glass scaffolds was also reported by direct laser spinning of glass fibers (Figure 3) [53].

Most bioactive glasses—including those doped with cobalt—contain silicon oxide as the major network forming, thus being characterized by relatively slow or even negligible dissolution over time unless they are produced as nano-sized powders. On the contrary, melt-derived biomedical glasses based on phosphorus pentoxide are soluble and have been widely studied as resorbable carriers for a number of therapeutic cations, including Ag^+^, Cu^2+^, Fe^3+^, Ti^4+^, etc., especially addressed to perform antimicrobial effects [54]. Phosphate glasses undergo dissolution in aqueous media (like biological fluids), and the resorption kinetics are dictated by glass composition [55]. However, there is a paucity of studies about Co-doped phosphate glasses, and just a couple of them was found in the literature. Peticone et al. [56] investigated the in vitro biocompatibility of phosphate glass microspheres doped with 5 mol.% TiO_2_ and 2–5 mol.% CoO using human osteosarcoma cell line MG-63 cultured under static and dynamic conditions in an orbital shaker. All glass compositions allowed cell proliferation and the formation of cell aggregates over a 2-week culture period. Neither at the highest concentration of cobalt was cytotoxic as cell metabolism was not altered. Interestingly, a higher agitation of the culture medium was associated with a decrease in cell growth. As expected, cobalt doping could induce VEGF upregulation in MG-63 cells, but no clear increase with increasing CoO content was observed.

Focusing on antimicrobial properties, Raja et al. [57] investigated the effects of a set of phosphate glasses doped or codoped with Co, Cu and Zn (up to 5 mol.% for each ion) against *S. aureus* (Gram-positive bacterium), *E. coli* (Gram-negative bacterium) and *C. albicans* (fungal strain) at a concentration of 5 mg/mL. A synergistic antimicrobial efficacy was observed in Cu/Co-codoped glasses against E. coli as compared with single-doped materials. Furthermore, the glasses containing the highest concentration of CoO (5 mol.%) exhibited minimal cytotoxicity towards osteoblast-like SAOS-2 cells compared with the dopant-free counterpart.

### 3.2. Sol-Gel Glasses

The first Co-doped bioactive glasses produced by the sol-gel process were synthesized in 2012 by Wu’s research team [41], who combined macro- and meso-templating strategies to fabricate glass scaffolds with hierarchical porosity. Specifically, they incorporated 2 to 5 mol.% of cobalt into a SiO_2_–CaO–P_2_O_5_ mesoporous bioactive glass (MBG) to partially replace calcium using co-templates of Pluronic P123 to produce an ordered mesoporous structure (mesopore size of 2–50 nm) and polyurethane foams to create large pores with a size of several hundred micrometers. The prepared Co-doped MBG scaffolds significantly enhanced VEGF secretion, HIF-1α expression and bone-related gene expression (ALP and osteocalcin) in bone BMSCs as compared with Co-free MBG scaffolds. However, the highest cobalt content caused reduced cell proliferation as compared with 2 mol.% Co-doped and Co-free MBG. These materials were also proposed as carriers for the controlled release of ampicillin molecules that were loaded in the mesopores: antibiotic-loaded scaffolds exhibited an antibacterial effect against *E. coli*, whereas drug-free Co-doped scaffolds did not, suggesting that the release of Co^2+^ ions alone—at least from this system—was unsuitable for antimicrobial purposes.

Studies on Co-doped MBGs were then discontinued—probably due to the high fragility of these scaffolds—and only after a hiatus of more than six years a new research work was published about the influence of cobalt incorporation (5 mol.%) and precursor selection on the structure and properties of 58S-derived sol-gel glasses [58]. When CoCl_2_.4H_2_O was used as a precursor, crystalline Co_3_O_4_ was formed, indicating that Co^2+^/Co^3+^ ions were not fully incorporated into the glass structure as network modifiers, whereas no crystals were observed if Co(NO_3_)_2_.4H_2_O was the precursor. The same research team, however, showed that if the amount of CoO was decreased to 2.5 mol.%, it could be fully incorporated as a network modifier in the CoCl_2_.4H_2_O-derived sol-gel glass [59]. In general, the addition of 5 mol.% cobalt slightly increased glass dissolution rate [58], in agreement with analogous results on melt-derived Co-doped glasses; more specifically, a higher release of cobalt was observed for the Co(NO_3_)_2_.4H_2_O-derived glass, and the released cobalt concentration was within the limits that induce pro-angiogenic effects. On the other hand, the incorporation of cobalt—regardless of the precursor used—reduced the apatite-forming ability over immersion for 2 weeks in SBF. However, if the amount of incorporated CoO was more moderate (2.5 mol.%), the effects on glass dissolution rate and apatite-forming ability became almost negligible, and no significant difference existed with respect to the Co-free sol-gel material [59].

**Figure 4:**
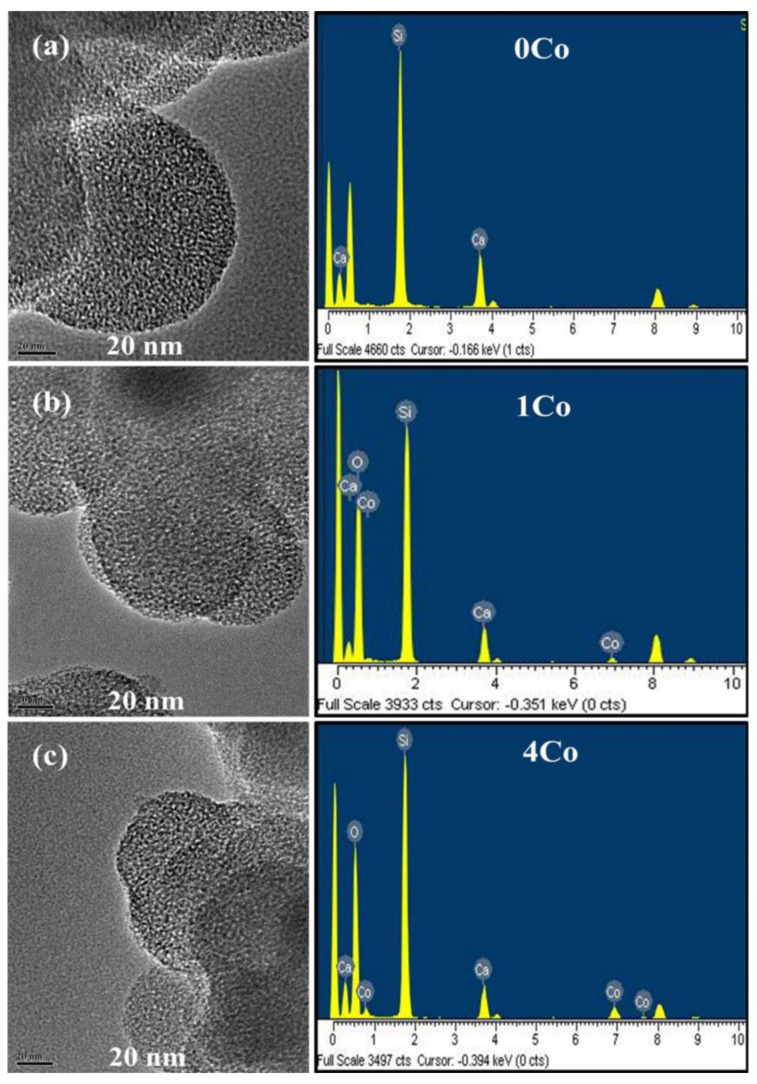
TEM images of bioactive silicate glass mesoporous nanoparticles without cobalt (**a**) and doped with 1 and 4 mol.% of CoO (**b**,**c**) along with the corresponding compositional analysis (EDS). Reproduced from [62] with permission.

If obtaining nanoscale materials is a goal, Co-doped silicate glass nanoparticles can be synthesized by a modified Stöber method followed by ultrasonication in Co(NO_3_)_2_.6H_2_O solution (to obtain binary SiO_2_–CoO glass from dense silica nanoparticles) [60], or ultrasonication in Co(NO_3_)_2_.4H_2_O/Ca(NO_3_)_2_.4H_2_O solutions (to obtain ternary SiO_2_–CaO–CoO glass from dense silica nanoparticles) [61], or else ultrasonication-assisted sol-gel method using cationic surfactant CTAB as a template for obtaining an ordered mesoporous structure [62]. These nanoparticulate systems (Figure 4) exhibited a controlled spherical morphology (50–120 nm) along with the sustained release of Co^2+^ ions, which was due to the significantly high specific surface area, especially in the latter case where a mesopore-templating agent was used (>700 m^2^/g) [62].

The presence of cobalt as a modifier in the glass network of mesoporous materials may interfere with the mesophase formation leading to a decrease in pore volume and specific surface [39]; however, Philippart et al. [63] revealed that if the amount of CoO in the glass composition is sufficiently low (<0.8 mol.%), incorporation of P_2_O_5_ can allow entrapment of Co^2+^ ions by PO_4_^3-^ groups, thereby preventing them from behaving as modifiers of the silica network and ultimately providing these glasses with higher network connectivity, mesoporous order and textural characteristics.

Glass nanoparticles could also be added as a bioactive phase within a soft polymeric matrix. In this regard, Grossi Santos de Laia et al. [64] embedded CoCl_2_.4H_2_O-derived 58S sol-gel glass particles in a poly(vinyl alcohol matrix) (PVA) to fabricate composite scaffolds by gel-cast foaming process.

In another study, PLGA/collagen electrospun nanofibers were rinsed in a suspension of Co-doped bioactive glass nanoparticles in distilled water (concentration of 0.1 *w*/*v*) and then freeze-dried to obtain the final fibrous composite scaffolds [65].

From the processing viewpoint, it should be noted that achieving a homogenous glass (nano)particle distribution inside the polymeric is a difficult task, especially at high filler ratios. In turn, particle agglomeration can yield mechanical problems due to stress concentration.

Fine powders of sol-gel bioactive glass doped with 2% to 5% of Co were also mixed with Pluronic F-127 to obtain a printable ink to be robocast in the form of grid-like scaffolds [66].

Some examples of Co-containing sol-gel glass products are listed in Table 1.

**Table 1:** Main characteristics of porous constructs containing Co-doped bioactive glasses or derived thereof.

Materials	Type of Product	Production Process	Morphology, Architecture and Mechanical Properties	Reference
Melt-derived Co-doped 13–93 glass (1–5 wt.% CoO)	Scaffold	Foam replication	Porous architecture (89–95 vol.%) with foam-like macropores; compressive strength 2.3–4.2 MPa	[37]
Melt-derived Co-doped glass (0.5 mol.% CoO) + PCL	Composite scaffold	3D printing	Grid-like architecture of oriented macropores with total porosity around 25 vol.%; compressive strength 2–4 MPa	[50]
Melt-derived Co-doped glass (4 mol.% CoO) + collagen/glycosaminoglycans	Composite scaffold	Freeze-drying	Highly porous composites (porosity 98 vol.%); compressive modulus from 1 to 5 MPa with increasing glass amount	[52]
Melt-derived Co-doped glass (5 mol.% CoO)	Single-phase fibrous glass mat	Laser spinning	Fibrous (cotton-candy) meshes	[53]
Co-doped sol-gel 58S glass (5 and 10 mol.% CoO) + PVA	Composite scaffold	Gel-cast foaming	Foam-like architecture with total porosity 46.9–68.5 vol.%	[64]
Co-doped sol-gel glass-ceramics (2–5% CoO)	Scaffold	3D printing	Grid-like architecture of oriented macropores; compressive strength 20.19 ± 7.06 MPa	[66]
Co-doped sol-gel 64S glass (5 mol.% CoO) + PLGA/collagen	Composite scaffold	Electrospinning + freeze-drying	Woven microstructure deriving from the electrospun polymeric fibers	[65]
Co/Cu-doped (5 wt.% CoO + 3 wt.% CuO) melt-derived glass in the Na_2_O–CaO–SiO_2_–TiO_2_–B_2_O_3_–P_2_O_5_ system + fish collagen	Composite mats	Electrospinning	Woven microstructure deriving from the electrospun polymeric fibers; tensile strength 2–4 MPa	[51]

## 4. In Vitro Biocompatibility

Cobalt-releasing bioactive glasses have been proposed as hypoxia-mimicking biomaterial to be used for the artificial stabilization of HIF-1α. The reason behind their application in biomedicine relies on the fact that Co can ultimately promote the hypoxia response, cell growth and angiogenesis, as revealed by the enhanced expression of HIF-1α and VEGF [67]. It was also shown that BMSCs pre-treated with CoCl_2_ could induce a higher degree of vascularization and osteogenesis in collagen scaffolds implanted in mice as compared with untreated implants [68]. More recently, Solanki et al. [53] did not observe such a rise in HIF-1α levels when CoCl_2_ was introduced to the conditioned media from Co-free glass. A similar trend was reported for VEGF expression, too, although the Co-free glass still stimulated VEGF expression compared with the DMEM control. This implies that the Co species released from the glass creates a synergistic effect with other glass-derived ionic dissolution products, which differs from the case in which CoCl_2_ is utilized as the cobalt source in the media. However, Co is toxic when applied at high dosages, and further investigation is needed to understand the mechanism through which cobalt released from glass activates the HIF pathway [69].

Therefore, not surprisingly, Hoppe et al. [70] confirmed—and quantified as well—that a dose-dependent trend exists for in vitro biocompatibility in the case of melt-derived Co-releasing 13-93-based glasses. They reported that glasses doped with 1 wt.% CoO was cytocompatible towards osteoblast-like MG-63 and endothelial cells, whereas the incorporation of 5 wt.% of CoO was cytotoxic to both cell types. This biological outcome was consistent with the results reported in a previous study showing that Co^2+^ ion concentrations of 2 ppm and 12 ppm were released from 13-93-derived glasses doped with 1 and 5 wt.% of CoO, respectively [37]. Accordingly, the safety window can be set in the range of 2 to 12 ppm, while the therapeutic window was suggested to be even narrower (5 to 10–12 ppm).

Co-doped glasses produced by the sol-gel process were also tested in vitro in terms of biocompatibility. Silicate gel-derived glasses containing 2.5 mol.% of CoO exhibited no cytotoxicity towards human adipose stem cells (hASCs) and human umbilical vein endothelial cells (HUVECs); furthermore, in vivo responses showed no adverse reactions and indicated the presence of newly formed vessels after bioactive glass implantation in the dorsum of rats, which was the proof of Co-stimulated angiogenesis [59]. These early results were corroborated in a further study on the same glass, which was shown to promote the formation of tubes and the gene expression of HIF-1α and VEGF-A both in vitro (HUVECs) and in vivo (rat dorsal region) [71].

On the other hand, sol-gel glasses doped with 1 mol.% CoO were more cytotoxic towards HUVECs as compared with Co-free glasses belonging to the same basic oxide system doped with 1 mol.% copper [34].

When glasses are produced in the form of SiO_2_–CoO sol-gel nanoparticles, no significant reduction in HUVEC viability was observed after 72 h for particle concentrations of 100 μg/mL and 500 μg/mL despite the sustained release of Co^2+^ ions, suggesting the suitability of these nanomaterials as pro-angiogenic implants for biomedical applications [60].

As regards Co-doped borate glasses, it was shown that 13-93B3 glass particles (size within 100–300 µm) doped with 1 wt.% cobalt did not decrease the viability but increased the homing capacity of adipose stem cells and bone marrow mesenchymal stem cells as compared with dopant-free control [46].

Cobalt-releasing glass particles were also incorporated inside soft matrices to produce composites; in this regard, Solanki et al. [72] reported that fibrous mats embedding 30 wt.% of melt-derived glass particles (size within 60–80 µm) in a matrix of electrospun polycaprolactone fibers did not reduce the metabolic activity of primary fibroblasts for up to 3 days when the cells were in contact with conditioned media from the composites. Therefore, the incorporation of glass particles in a polymeric matrix is useful for reducing cytotoxicity while maintaining an adequate functional effect (promotion of VEGF secretion).

## 5. Applications

### 5.1. Bone-Contact Field

Cobalt-doped bioactive glass particles alone or embedded in polymeric matrices (e.g., collagen), thereby producing “soft” composites, have been proven to create a microenvironment capable of stimulating both angiogenesis and vascularisation via the release of cobalt, according to the hypoxia-mimicking mechanism, as well as supporting osteogenesis as a result of osteoinductive ion release from glass [52]. Suitability for bone regeneration has been convincingly proved in many studies in vitro and in vivo.

**Figure 5:**
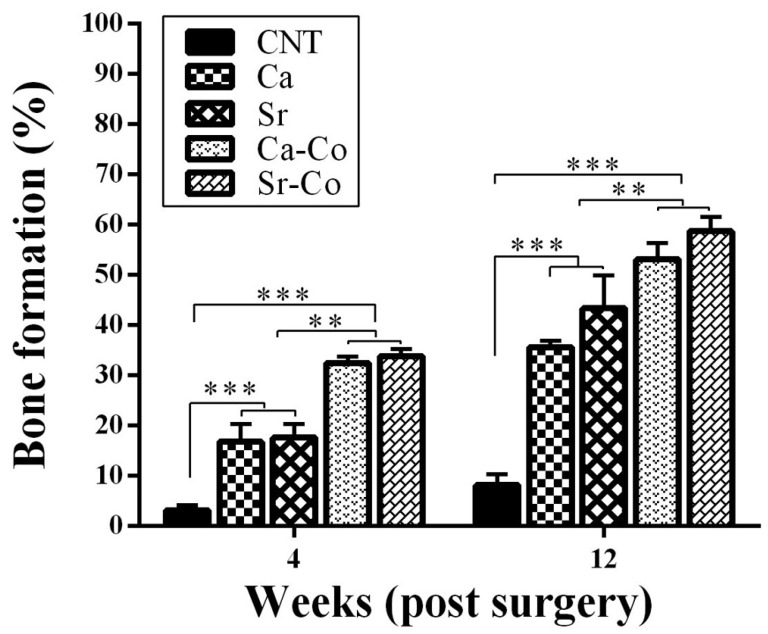
Histomorphometric analysis showing the percentage of new bone formation around glass granules of different compositions implanted in rabbits (** *p* < 0.01, *** *p* < 0.001). Reproduced from [78] with permission.

Furthermore, bioactive glass codoping with cobalt and strontium has been proposed as highly appealing because the latter exhibits antiresorptive properties through the reduction of osteoclast activity and has been used for many years in the form of strontium ranelate in the treatment of osteoporosis [73]. In this regard, a research team coordinated by Prof. Hill deeply studied the osteogenic effect of a series of melt-derived multicomponent silicate glasses doped with 0.5 mol.% CoO and/or 6 mol.% SrO both in vitro and in vivo. A first report focusing on glass particles with a size below 38 µm confirmed the apatite-forming ability of all the glasses in SBF as well as their osteogenic potential using SAOS-2 cells, as revealed by ALP testing and calcium nodule formation, although the ameliorative effect of Co/Sr-codoping as compared to Sr-doping was not so apparent [74]. The same in vitro results were substantially confirmed in a second study, where the particle size effect was also evaluated; specifically, glass particles with finer size (9 μm) were found to be more effective than larger particles (725 μm) in stimulating osteogenic gene expression [75]. On the other hand, it was reported that the smaller particles had higher cytotoxicity against SAOS-2 cells as compared with the larger ones: this can be related to the higher surface area of the formers—associated with higher ion release with potentially more toxic but also more osteo-stimulatory effects—and further demonstrates the importance of both composition and particle size for the postoperative success of bioactive glass implants in a particulate form. The same glass compositional systems with particle size below 38 μm were also tested using induced pluripotent stem cells (iPSCs), which offer great promises for osseous repair and regeneration that cannot be achieved with other cell sources (e.g., embryonic stem cells), including the absence of ethical issues, the lack of immune rejection as well as the ability to differentiate into osteogenic cells [76]. Although incubation of iPSCs with such glasses resulted in a significant reduction of cell viability over 7 days because of cell apoptosis, the data obtained from ALP activity assay and gene expression revealed that the iPSCs could adhere and spread onto the glass particles and over-express the osteogenic markers, including osteocalcin, osteonectin and Runx2 [77]. The same glass compositions were eventually tested in vivo: after being seeded with HUVECs, glass granules in the range of 100 to 1000 μm were implanted in critical size defects of distal femur in rabbits and, according to histologic and immune-histologic results, bone healing was better in the group receiving Sr/Co-codoped glass constructs in comparison with the Co-free groups at both 4 and 12 weeks of follow-up (Figure 5) [78]. These Co/Sr-codoped glasses were also produced by the sol-gel process by Kermani et al. [79], who reported similar results to the melt-derived counterparts in vitro in terms of cytocompatibility with osteoblast-like MG-63 cells, calcium nodule formation and a pro-angiogenic effect with HUVECs despite the more significant (and potentially toxic) ionic release due to the higher specific surface area of mesoporous glass particles.

While these studies provide convincing evidence of the unique beneficial effect carried by the presence of cobalt over other elements (e.g., strontium used alone), this has not always been clearly demonstrated in other reports. For example, although Co-releasing sol-gel glass clearly stimulated the secretion of VEGF in HUVECs compared with the Co-free glass control, this effect was significantly lower than that elicited by the same glass doped with the same amount of copper (1 mol.%) [34]. In another study, Co/Ag/Ti-multidoped 58S-derived glass coatings on titanium were proposed for improving osteointegration and reducing bacterial contamination in bone implants, but the actual superiority of Co-containing material compared to glasses doped with only Ti^4+^ or Ag^+^ ions did not clearly emerge [80].

A synergistic effect between cobalt and boron co-released from melt-derived 45S5 glass particles (size below 25 µm) was reported by Chen et al. [81] in increasing the VEGF secretion from bone marrow-derived stromal ST-2 cells.

One study also suggested potential application in dental tissue engineering as melt-derived Co-doped silicate glass particles (1 mol.% CoO, size below 38 μm) simulated the secretion of VEGF in dental stem cells [82].

Besides being proved to be ideal biomaterials for bone regeneration, bioactive glasses have been reported to stimulate chondrocyte activity as well, thus showing promise for the repair of osteochondral defects at the bone–cartilage interface [83,84]. Reduced oxygen tension was observed to promote chondrogenic differentiation [85]; it was also suggested that enhanced chondrogenesis in low-oxygen environments is primarily mediated through HIF-1α by inducing the expression of pro-chondrogenic genes such as Sox9 [86]. Starting from the hypothesis that activation of the HIF-1α pathway could enhance the differentiation of hMSCs into chondrocytes, Littmann et al. [36] investigated whether melt-derived Co-doped bioactive glasses (1 to 2 mol.% CoO, particle size below 38 µm) could actually promote chondrogenesis. Interestingly, results showed that continued exposure to the Co-containing glass extracts significantly reduced hMSC proliferation, viability and chondrogenic differentiation, thus suggesting caution in application for cartilage regeneration.

Most uses of Co-releasing glasses in contact with the bone are directly related to improving tissue regeneration; however, it is worth mentioning another functional application addressed to radiation shielding (e.g., gamma ray or neutron attenuation), which deserves to be considered for diagnostic and theranostic purposes [87,88,89].

### 5.2. Cancer Treatment

Some special types of implantable glasses have been proposed since the 1980s for the treatment of selected cancers. In the case of hepatocellular carcinoma, therapy based on an arterial infusion of radioactive aluminosilicate glass microspheres with 25 µm diameter was FDA-approved and cleared for clinical use in 1999. However, most research dealing with bioactive glass and cancer is addressed in the field of hard tissues. In this regard, the relevant strategies involving bioactive glasses and glass-ceramics typically refer to radiotherapy (via doping with radioactive elements) [90], drug delivery (through acting as carriers for chemotherapeutics, such as in the case of mesoporous glasses) [91], magnetic hyperthermia (by embedding magnetic crystalline phases in the glass to develop heat upon exposure to an external magnetic field) [92] and photothermal therapy (where the dopant in the glass network can absorb near-infrared (NIR) light of a laser and convert it into heat) [93]. The last two options are of significance for Co-doped glasses and rely on the fact that cancer cells are more sensitive than healthy cells to temperature increments up to around 43 °C, which yield a generation of reactive nitrogen/oxygen species in the patient’s body to locally destroy malignant cells [94].

To the best of the authors’ knowledge, at present, just two studies have been specifically addressed to proposing Co-doped bioactive glasses to combat cancer. The first one refers to potentially applying magnetic hyperthermia by using 45S5 glass/hydroxyapatite composites containing Fe_2_O_3_ and CoO, but no evidence of the functional effect in vitro or in vivo has been reported yet [95]. As regards the photothermal strategy, Liu et al. [66] prepared a series of gel-derived SiO_2_–CaO–P_2_O_5_ mesoporous glasses doped with 5 mol.% of Co, Cu, Mn and Fe and investigated their antitumor potential under NIR-irradiation in vitro and in vivo after subcutaneous injection of osteosarcoma SAOS-2 cells in mice. The tumor tissue necrosis rate associated with Co-doped glass was significantly lower compared with the other doped materials and comparable to Co-free glass. Although the authors claimed that “the prepared doped scaffolds possessed excellent photothermal performance”, the reported results actually suggest that the choice of cobalt is not the best one for this specific therapeutic approach.

On the other hand, cobalt can be more successfully involved in other implantable bioceramic systems (e.g., cobalt-ferrite nanoparticles) in different cancer treatment strategies, such as magnetic hyperthermia [96].

We should also consider that photothermal therapy mediated by implanted glasses is recommended for superficial malignant lesions (e.g., on the skin), as living tissues can absorb the energy of the NIR beam. Therefore, this therapeutic approach has some inherent restrictions in penetration depth that should not exceed 1 cm to ensure adequate efficacy [97], and thus the treatment of deep solid tumors such as bone cancer remains a challenge unless the light may be somehow brought to the injured site (e.g., by using special optical fibers).

### 5.3. Wound Healing

Given the potent pro-angiogenic effect induced by Co^2+^ ions, it has been recently proposed that the use of Co-releasing biomaterials for the treatment of skin lesions or, more generally, wound healing, where vascularization indeed plays a key role in achieving a positive clinical outcome. The first report that specifically addressed this goal was published in 2017 by Moura et al. [98], who fabricated composite mats comprising polycaprolactone electrospun fibers and Co-doped sol-gel glass nanoparticles (2.5 mol.% CoO). The in vitro acellular studies showed that mineralization in SBF took place if the glass nanoparticle concentration exceeded 0.75 wt.% in the composite. This is a matter of great concern for wound healing applications. The use of bioactive glasses in this specific field is highly challenging because it is in apparent contrast with the inherent, most famous property of these materials, i.e., the apatite-forming ability upon immersion in body fluids. In fact, hydroxyapatite formation was reported to inhibit hemostasis [99], and calcium deposits have been shown to delay or even stop the healing of leg ulcers [100]. Therefore, new bioactive glass compositions had to be developed so that no calcium phosphate layer forms on the material surface; in this context, the term “bioactive” does not refer to hydroxyapatite formation but, rather, to the stimulation of a beneficial biological response through ionic species release—in this case, enhanced angiogenesis promoted by Co^2+^ ions. Profs. Stevens’ and Jones’ research teams first tried tackling this challenge by designing ad-hoc glass compositions where P_2_O_5_ was not included, and CaO was replaced by MgO in an attempt to minimize calcium phosphate formation so that Co^2+^ ions could be released during glass dissolution without the precipitation of any hydroxyapatite layer [101]. This objective was achieved by keeping the network connectivity close to that of 45S5 Bioglass^®^ (2.12), which is known to undergo dissolution both in vitro and in vivo. Some of the melt-derived glasses produced in that study still exhibited mineralization ability in SBF, and after careful investigation, the composition 50SiO_2_–24Na_2_O–24MgO–2CoO (mol.%) was selected for cellular studies because it showed negligible calcium phosphate deposition and sustained cobalt release. Biological in vitro tests revealed that the conditioned media from fibrous composites embedding particles of this glass inside an electrospun polycaprolactone matrix actually stabilized HIF-1α and significantly increased the expression of VEGF in human primary fibroblasts, which are indeed involved in dermal regeneration.

The same research group used another of these previously developed compositions (55SiO_2_–20Na_2_O–10K_2_O–10MgO–5CoO (mol.%), which was found able to release Co^2+^ ions into SBF without forming a calcium-phosphate layer over 21 days, for producing glass fibers by laser spinning [72]. The in vitro biological tests with human dermal keratinocytes exposed to fiber-conditioned media revealed that this glass activated the HIF pathway and promoted the expression of VEGF in such cells.

**Figure 6:**
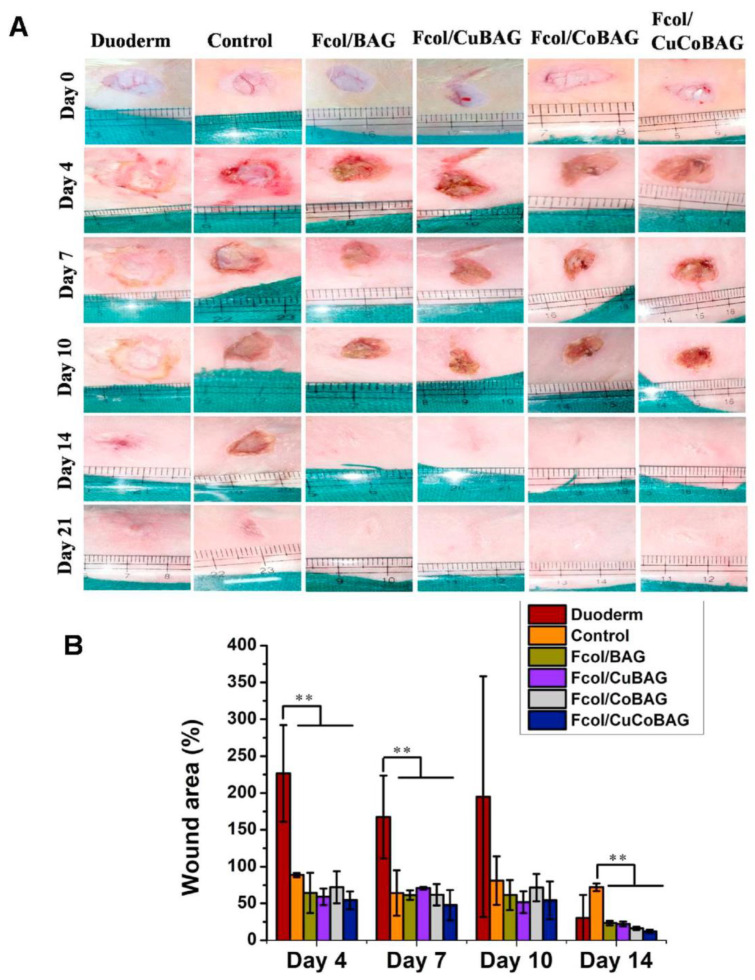
Results from in vivo tests in diabetic rabbits: (**A**) full-thickness skin wounds in untreated (control) and treated groups receiving four types of microfibrous mats or a commercial dressing (Duoderm); and (**B**) graphical representations of the wound area (%) over time (** *p* < 0.01). Reproduced from [101] with permission.

The effects and potential of glass codoping with cobalt and copper were recently investigated in vitro and in vivo by Jana et al. [51], who prepared composite mats based on electrospun fibers of collagen extracted from Rohu fish skin and melt-derived bioactive glass particles doped with cobalt (5 mol.%) and copper (3 mol.%). After being implanted in the dorsum of rabbits, these microfibrous constructs induced better neovascularization, re-epithelization and ordered deposition of ECM components such as collagen and elastin compared with commercial wound dressing used as a control. Enhanced wound healing ability was attributed to the synergic effect of the ionic dissolution products (Cu^2+^, Co^2+^ and other ions) from the bioactive glass along with fish-derived collagen, which collectively accelerated the sequential steps of the skin healing process. A subsequent study in rabbits showed that these microfibrous constructs were effective in enhancing cutaneous wound healing under diabetic conditions, too (Figure 6) [101].

A synergistic effect of cobalt with zinc on wound closure efficacy as compared with the effect of single ions was also suggested by Vinyak et al. [102], who treated full-thickness injuries in rabbits by using Zn/Co-codoped glass-coated eggshell membranes. Recently, Souza et al. [103] have tried to incorporate Ce and Co into a mesoporous SiO_2_ network through a series of steps, including a reaction with cobalt/cerium nitrate solutions, followed by incubation, sonication, centrifugation, air-drying and ultimately a calcination process at 680 °C for 3 h. While they were able to detect the incorporation of Co and Ce in the SiO_2_ network, we question the reproducibility of this method, as the resulting materials exhibited significant uncontrolled crystallization, rendering them unreliable. Moreover, the synthesized materials only released a maximum of 0.6 ppm of Ce (in 3 days) and 2 ppm of Co (in 8 days), which falls below the therapeutic range. However, these materials demonstrated catalase-mimetic activity, suggesting their ability to scavenge reactive oxygen species generated by hydrogen peroxide. Antibacterial behavior was observed at high concentrations, and the materials biocompatibility with cells in vitro was confirmed through flow cytometry. The codoping of Co with other antibacterial elements, such as cerium, is an interesting approach deserving further exploration, provided that an appropriate experimental methodology is employed [103].

## 6. Conclusions and Outlook

The currently available papers published from 2010 to date about Co-releasing bioactive glasses (about 50 studies specifically focused on this topic) have been reviewed in this article: on the one hand, this amount of the literature witnesses the interest of the glass community towards such biomaterials but, on the other hand, reveals the concerns behind their use.

Evidence has been reported that the addition of cobalt to melt-derived bioactive glass can delay material dissolution, ion release and hydroxyapatite formation in a concentration-dependent manner [52]. If the Co-doped glass is produced through the sol-gel method, cobalt precursor was shown to play an important role in defining the final glass structure, dissolution kinetics and reactivity [59] and should therefore be carefully evaluated in the context of tissue engineering applications.

In contrast to many other ions, the supplementation of cobalt is not performed to directly stimulate osteogenesis but rather for pro-angiogenic purposes. In fact, cobalt was shown to inhibit osteoblast proliferation and ALP gene expression. Hence, cobalt is a promising therapeutic ion because its pro-angiogenic effect counterbalances its possibly negative osteogenic impact. It is worth highlighting that combined supplementation of cobalt and other ions having significant osteogenic properties may be beneficial for successful bone tissue regeneration [78]. In this regard, cobalt can provide the required angiogenic environment to indirectly potentiate the inherent osteogenic properties of bioactive glasses.

Because of the potent pro-angiogenic effect of cobalt, there is convincing evidence about the suitability of Co-releasing bioactive glasses for promoting wound healing and potential skin regeneration, while limited applicability has been shown so far for photothermal therapy in the context of cancer treatment. According to the target application, the network connectivity can be properly designed, taking into account the addition of CoO in order to ultimately tailor the extent of apatite-forming ability [72]. While this property is indeed desirable for stimulating bone regeneration, it is not advisable in contact with soft tissues (e.g., dermal regeneration).

The abundant literature on the biological effects elicited by metallic ions on the activity of various types of cells has demonstrated that even some “exotic” elements of the periodic table, at proper dosages, may have apparently therapeutic or beneficial properties. In the field of bioactive glasses, this point was well underlined in a recent review by Boccaccini’s group [24]. Cobalt, however, is known to induce important toxic effects in the body also at relatively low dosages. Therefore, a crucial question to which scientists are asked to give a more definite response is, can the incorporation of cobalt in bioactive glasses be fully justified by the advantages over the potential risks on the basis of a frank and scientifically defined cost–benefit ratio? Hence, if the response is not unanimous, a second key question is, can the beneficial effect of cobalt be achieved to the same extent by using another “safer” element? At present, some studies seem to suggest the answer “yes” to the latter question—for example, a comparable effect in terms of cell viability and osteogenic differentiation in vitro was obtained by doping glasses only with strontium rather than strontium and cobalt [74]—but long-term trials are needed to achieve a truly definite conclusion. In order to save experimental time with associated ethical issues and costs, the implementation of computational machine-learning strategies could be helpful in this regard [104]. Incorporation of small amounts of Co-doped bioactive glass as a “therapeutic phase” inside a polymeric matrix also seems a valuable approach to minimize the side effects, along with benefitting from other advantages such as pliability, softness and easy shapability during surgery.

Providing an honest and scientifically supported response to the above-mentioned questions is more than ever necessary to understand whether Co-releasing bioactive glasses are really a hope for many branches of tissue engineering and regenerative medicine or if they are a mere, albeit fascinating, hype.

## Data Availability

This paper is a review; therefore, data are available in the source publications listed in the bibliography.
